# Development of a dual-target RAA-LFD assay for point-of-care and visual detection of *Salmonella* pullorum and *Salmonella* typhimurium in fecal samples

**DOI:** 10.3389/fvets.2025.1684537

**Published:** 2025-11-03

**Authors:** Weiye Zuo, Congxue Shao, He Qin, Hongwei Gao, Xuemei Sun, Pengyan Wang, Jingjing Ren

**Affiliations:** 1 Laboratory of Animal Immunology Engineering, College of Animal Science and Technology, Shihezi University, Xinjiang, China; 2 Xinjiang Taikun Group Co., Ltd., Xinjiang, China

**Keywords:** *Salmonella* pullorum, *Salmonella* typhimurium, recombinant enzyme-assisted amplification, lateral chromatography test strips, visual detection

## Abstract

To address the need for rapid detection of *Salmonella* pullorum (*S.* pullorum) and *Salmonella* typhimurium (*S.* typhimurium) in the poultry industry, we developed a dual-target point-of-care system integrating recombinase-aided amplification (RAA) with lateral flow dipsticks (LFD) for visual pathogen identification (RAA-LFD). Using primers and probes specifically targeting the *ipaj* gene of *S.* pullorum and the *STM4497* gene of *S.* typhimurium, the optimized assay achieved detection at 37 °C within 20 min. The dual RAA-LFD assay showed exceptional specificity with no cross-reactivity toward non-target pathogens. Detection sensitivities reached 5.91 × 10^1^ CFU/mL (*S.* typhimurium) and 2.37 × 10^2^ CFU/mL (*S.* pullorum) in pure cultures. In contrast, genomic DNA detection achieved identical limits of 5.70 × 10^1^ fg/μL (*S.* typhimurium) and 4.53 × 10^1^ fg/μL (*S.* pullorum). In artificially contaminated samples, the detection limits reached 3.92 × 10^2^ CFU/mL for *S.* pullorum and 6.26 × 10^1^ CFU/mL for *S.* typhimurium. Clinical validation demonstrated 96.88–100% coincidence with biochemical identification and multiplex PCR results. This study confirms the precision and high sensitivity of the dual RAA-LFD assay as a detection methodology. Furthermore, by eliminating reliance on complex traditional techniques, this technology provides an efficient grassroots-level field screening tool with significant potential for preventing avian salmonellosis and enhancing food safety monitoring.

## 1 Introduction

*Salmonella*, classified within the Enterobacteriaceae family, is Gram-negative and exhibits rod-shaped morphology (1). It comprises over 2,600 serotypes globally, and these pathogens cause more than 100 million avian deaths annually (2, 3). Among poultry-specific serovars, *S.* typhimurium and *S.* pullorum are the predominant ones, causing salmonellosis fever and pullorum disease, respectively (4, 5). Transmission occurs primarily via fecal-oral contamination of water or feed (6, 7). *S.* pullorum and *S.* typhimurium pose a significant annual economic burden on poultry production in developing countries where standardized prevention and control facilities are lacking (8, 9). Therefore, the timely detection of *S.* typhimurium and *S.* pullorum in poultry feces is crucial for maintaining poultry health and controlling disease transmission.

The traditional White-Kauffmann-Le Minor scheme remains the gold standard for Salmonella serotyping. However, this method is time-intensive and costly (10). Nucleic acid amplification approaches, such as polymerase chain reaction (PCR) and loop-mediated isothermal amplification (LAMP), exhibit heightened analytical sensitivity compared to traditional serological methods. However, PCR requires thermal cycling equipment and skilled personnel, limiting its field applicability. While LAMP offers rapid, instrument-free detection, it suffers from high false-positive rates due to risks of aerosol contamination, which complicates on-site implementation (11). RAA, an optimized derivative of recombinase polymerase amplification (RPA) isothermal amplification technology, enables rapid diagnosis of human and animal infectious diseases (12, 13). RAA mimics *in vivo* DNA replication to amplify target fragments under isothermal conditions. Key advantages include completion within 30 min at low temperatures (35–38 °C) (14) and approximately 50% lower cost than RPA (15). LFD generates labeled amplification products using labeled primers (16, 17). It employs a dip-strip antibody combination to facilitate rapid visual interpretation of successful amplification without the need for specialized instruments (18–21).

In this study, we targeted the *STM4497* gene of *S.* typhimurium and the *ipaj* gene of *S.* pullorum, designing three primer pairs for pathogen detection. Following optimization of RAA amplification parameters, we established a dual RAA-LFD assay for simultaneous detection of both pathogens. This method maintains the specificity and sensitivity, while streamlining procedures and reducing reagent consumption. Its rapid visual readout (20 min), minimal equipment requirements (37 °C incubation only), and workflow simplicity offer transformative potential for field diagnostics, mass screening programs, and resource-limited settings where timely *Salmonella* detection is critical.

## 2 Methods and materials

### 2.1 Bacterial strains, clinical samples and reagent

The bacterial strains detailed in Table 1, including reference controls *S.* pullorum and *S.* typhimurium, were selected to optimize dual RAA-LFD reaction conditions, determine detection sensitivities, and evaluate assay specificity. We collected 32 clinical fecal samples from diseased chickens in different regions of Xinjiang Province and stored them at −80 °C for subsequent analysis.

**Table 1:** Information on bacterial strains used for specific detection.

Species	Strain number	Source
*Salmonella* pullorum	BNCC273132	Beina Biological Co., LTD
*Salmonella* pullorum	SP2-22	Preserved in our laboratory
*Salmonella* pullorum	SP2-38	Preserved in our laboratory
*Salmonella* typhimurium	ATCC14028	ATCC
*Salmonella* typhimurium	Z5	Preserved in our laboratory
*Salmonella* typhimurium	Z9	Preserved in our laboratory
*Salmonella enteritidis*	GDMCC1.345	Preserved in our laboratory
*Escherichia coli*	ATCC25922	ATCC
*Pseudomonas putida*	KT2442	Preserved in our laboratory
*Enterococcus fecalis*	GDF22P19–1	Preserved in our laboratory
*Listeria monocytogenes*	ATCC19115	ATCC

ATCC, American Type Culture Collection; GDMCC, Microbial Culture Preservation Center of Guangdong Province, China.

DNA isothermal nucleic acid amplification kit (basic type) and colloidal gold dip-type isothermal nucleic acid amplification kit (RAA-LFD type) were purchased from Leshang Biotechnology Co., Ltd. (Wuxi, China). Lateral flow test strips (LFD) were purchased from Tiosbio Co., Ltd. (Beijing, China). Proteinase K and LB medium were purchased from Beijing Solaibao Technology Co., Ltd. DNA marker S10928, lysis buffer, squishing buffer, and 2 × Taq MasterMix (Dye Plus) were purchased from TransGen Biotechnology Co., Ltd. (Beijing, China), and DNA Extraction Reagent (Phenol: chloroform: isopentanol = 25:24:1) was purchased from Beijing Biolaibo Technology Co., Ltd.

### 2.2 DNA extraction

**Figure 1:**
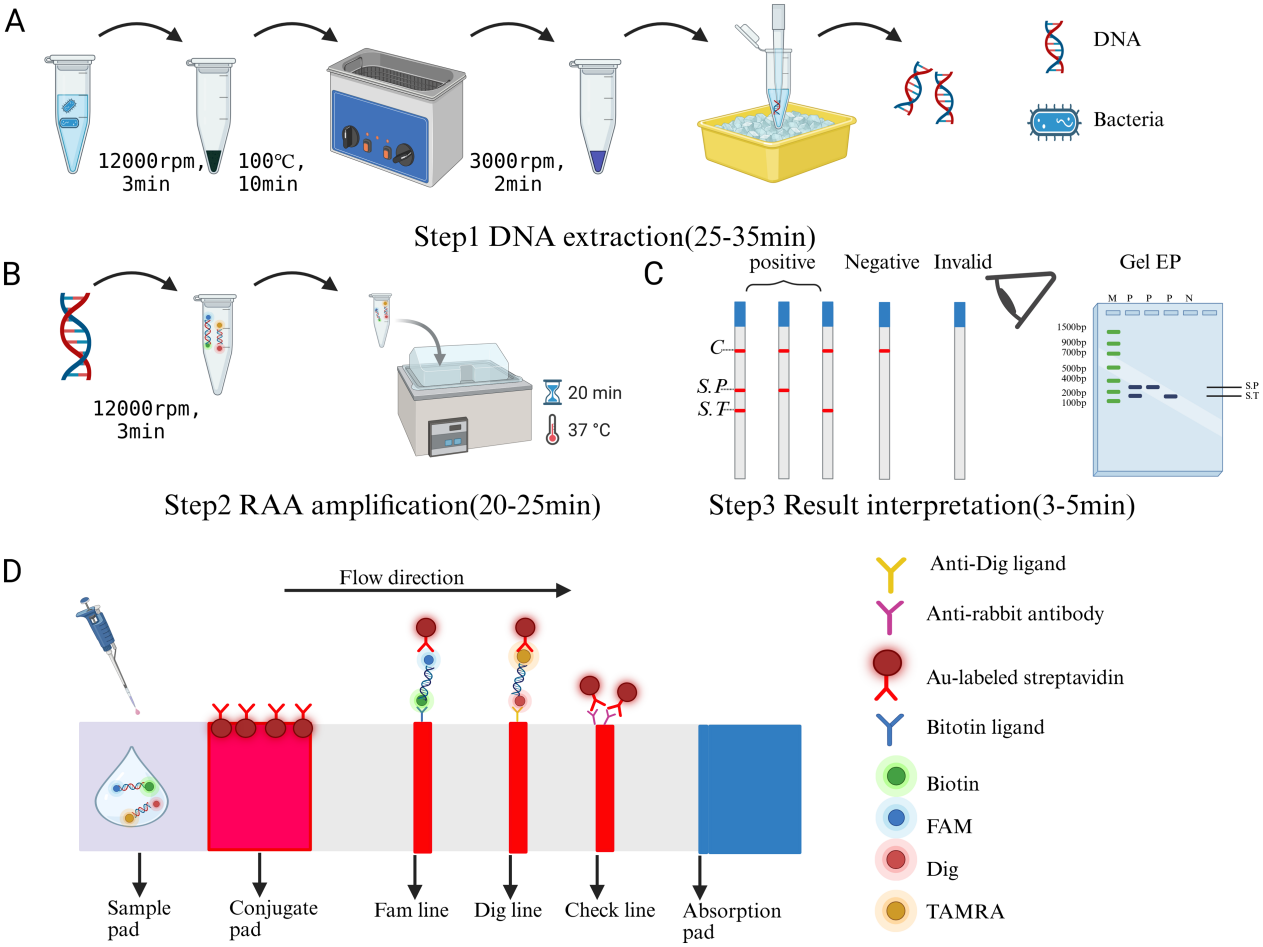
The working principle and steps of dual RAA-LFD detection. **(A)** Step 1: Extract the DNA of *S.* pullorum and *S.* typhimurium by boiling in water. **(B)** Step 2: Perform dual RAA amplification using RAA primers and probes to produce biotin and FAM, or Dig and FamRA-labeled DNA amplification products. **(C)** Step 3: Analyze the amplification product by agarose gel electrophoresis or LFD. SP, *S.* pullorum; ST, *S.* typhimurium. **(D)** The working principle of LFD. Created in BioRender. Dwad, D. (2025) https://BioRender.com/19ezigy.

In this study, we used a simple boiling method for further DNA extraction (22). Cultures were prepared by inoculating 10 μL glycerol stocked strains into 2 mL broth (16 h, 37 °C, 250 rpm). After centrifugation of 1 mL culture (12,000 rpm, 3 min), the supernatant was discarded and the pellet was boiled in 50 μL medium (100 °C, 10 min). We centrifuged the heated tubes at 3000 rpm for 2 min and placed them on ice for 5 min (Figure 1A). Finally, we transferred the supernatant containing the DNA to a new tube and stored it at −20 °C.

### 2.3 Design of RAA primers and probes

Gene sequences for *S.* typhimurium *STM4497* (GenBank: NC_003197.2) and *S.* pullorum *ipaj* (GenBank: ADF43835.1) were retrieved from NCBI. Corresponding RAA primers and probes were designed using Primer Premier 5.0 following the RAA-LFD isothermal amplification kit specifications (RAA-LFD type). Oligonucleotide probes were designed with a length of 46–52 bp, featuring ≥30 nucleotides at the 5′ terminus and ≥15 nucleotides at the 3′ terminus. The 5′ end was conjugated to the FAM fluorophore. There was a base gap of 30 bp from the 5′ end of a probe, and the gap was altered with a methylhydrofuran residue, and the 3′ end was modified with a blocking group. The other investigation was modified with TAMRA at the 5′ end, a methylhydrofuran residue at the middle Nick, and a blocking group at the 3′ end. Designed primers measured 30–35 bp length and possessed 30–70% GC content. The amplicon length ranged from 100 to 300 bp, and Tm values were not required for this analysis. Primer specificity was confirmed via NCBI BLAST analysis and experimental screening to identify primers with maximal amplification efficiency. The best selected downstream primers 5′ of *S.* typhimurium and *S.* pullorum were labeled with biotin and digoxin, respectively (Table 2). The multiple PCR primer combinations *ipaJ-F/R*, *lygD-F/R*, and *mdh-F/R*, developed according to reference (23), were used as experimental controls for subsequent multiple PCR. Table 2 details all primers and probes. Oligonucleotide synthesis was conducted by Sangon Biotech (Shanghai).

**Table 2:** Primers and probes used in the *S.* pullorum and *S.* typhimurium dual RAA-LFD assays.

Primers and Probe	Sequence (5′-3′)	Product length (bp)	function	Targeted serovar
ST-F1	5′-TGTGGTCCTTTTCCAGATTACGCAACAGATAC-3′	159 bp	RAA	ST
ST-R1	5′-TGTCACAGGTTCAGAGCCGCATTAGCGAAGAG-3′			
ST-F2	5′-TGTGGTCCTTTTCCAGATTACGCAACAGATA-3′	158 bp	RAA	ST
ST-R2	5′-GTCACAGGTTCAGAGCCGCATTAGCGAAGAG-3′			
ST-F3	5′-CGAACTTGTGGTCCTTTTCCAGATTACGCAACA-3′	179 bp	RAA-LFD	ST
ST-R3	5′-Biotin-GCTTGAATACCGCCTGTCACAGGTTCAGAG-3′			
ST-probe	5′-6-FAM-CTCATTCTGAGCAGGATAATCAAAAATCCA[THF]AACCCAATCTCATTACCG-C3-spacer-3′			
SP-F1	5′-GTGCTTTTACTTCTGGGTACAGCCAAGATAAT-3′	151 bp	RAA	SP
SP-R1	5′-GATAGTTGTAGTAACCTAGCCGACGCTGGT-3′			
SP-F1	5′’-GTGCTTTTACTTCTGGGTACAGCCAAGATAAT-3′	151 bp	RAA	SP
SP-R2	5′’-GCCTTAACTAACGAATGTGAATCTGATTTGTA-3′			
SP-F1	5′’-GTGCTTTTACTTCTGGGTACAGCCAAGATAAT-3′	214 bp	RAA-LFD	SP
SP-R3	5′’-Dig-GCCTTAACTAACGAATGTGAATCTGATTTGTATAAA-3′			
SP-probe	5′-TAMRA-AAGATTTTTCTCCTCAGTAACATCGCAGCC(THF)ATTCCCAAAAGCCTGCAT-C3 Spacer-3′			
ipaJ-F	5′’-CTGTCTGCTGCCGTGAT-3′	633 bp	PCR	SP
ipaJ-R	5′’-GCACCCAGTGTAATCCAAC-3′			
lygD-F	5′’-CATTCTGACCTTTAAGCCGGTCAATGAG-3′	339 bp	PCR	SE
lygD-R	5′’-CCAAAAAGCGAGACCTCAAACTTACTCAG-3′			
mdh-F	5′’-TTCCACCACGCCCTTC-3′	505 bp	PCR	ST
mdh-R	5′’-GCCGGGTATGGACCGTTC-3′			

F, forward primer; R, reverse primer; P, probe; Dig, Digoxigenin; 6-FAM,6-Carboxyfluorescein; THF, tetrahydrofuran; C3-spacer 3/ -block; TAMRA, Tetramethylrhodamine; SP, *S*. pullorum; ST, *S*. typhimurium; SE, *Salmonella enteritidis*.

### 2.4 The establishment of dual RAA assays and dual RAA-LFD assays

The establishment of dual RAA assays: Dual RAA assays were developed using the basic nucleic acid amplification kit per manufacturer specifications, with slight optimization of reaction parameters. The 50 μL reaction mixture contained, in short, 25 μL buffer, 1.5 μL each of *S.* typhimurium primers, 1.5 μL each of *S.* pullorum primers, 12 μL ddH_2_O, and 2 μL of each DNA template. After thoroughly mixing, the mixtures were added to the reaction tube containing lyophilized enzyme powder. The mixtures were homogenized by finger-flicking at 3000 rpm for 2–3 s, and initiated by adding 3 μL magnesium acetate to the tube lids. Following secondary centrifugation, isothermal amplification was performed at 37 °C for 20 min. Products were extracted using DNA extraction buffer (50 μL), vortex-mixed (15 s), and pelleted (12,000 rpm, 2 min). The supernatant was removed, and the amplification products underwent 2% agarose gel analysis, using nuclease-free distilled water as a negative control.

The establishment of dual RAA-LFD assays: The RAA nucleic acid amplification kit (strip type) was used for RAA reaction strictly according to the instructions, and minor system modifications were made. In short, 25 μL buffer, 1.5 μL each of *S.* typhimurium primers, 1.5 μL each of *S.* pullorum primers (the primers based on dual RAA assays), 0.6 μL each of 10 μM ST/SP-probe, 10.8 μL ddH_2_O. After mixing 2 μL of each *S.* typhimurium and *S.* pullorum DNA template, they were added to the lyophilized RAA precipitate. The mixture was then mixed by flicking the finger and centrifuged at 3000 rpm for 2–3 s. Subsequently, a 3 μL aliquot of Mg(OAc)₂ solution was aliquoted into the inside of the tube cover. The mixture was reacted at a constant temperature of 37 °C for 20 min (Figure 1B). Finally, after the RAA product was diluted 10 times with ddH_2_O, 10 μL of the RAA reaction dilution liquid was added to the LFD pad. Results were interpreted by direct visual inspection after 3–5 min of incubation, with nuclease-free ddH_2_O as a negative control. The T1 line was colored if *S.* pullorum was present, the T2 line was colored if *S.* typhimurium was present, and both T1 and T2 lines were colored if both bacteria were present (Figures 1C,D).

### 2.5 Optimization of dual RAA-LFD assay conditions

Based on the dual RAA-LFD assays established in section 2.4, the optimal primers for *S.* pullorum and *S.* typhimurium were determined, respectively. Subsequently, primer concentration screening was performed. For *S.* typhimurium primer screening: A fixed concentration (10 μM) of *S.* pullorum primers was used with gradient concentrations (10, 8, 6, 4, 2, and 1 μM) of *S.* typhimurium primers, followed by the addition of 2 μL *S.* typhimurium DNA. For *S.* pullorum primer screening: The optimized *S.* typhimurium primer concentration was applied with gradient concentrations (10, 8, 6, 4, 2, and 1 μM) of *S.* pullorum primers, followed by 2 μL *S.* pullorum DNA. All reactions underwent incubation at 38 °C for 20 min, optimal concentrations were identified via LFD band analysis.

With the optimal primer concentrations established, the effect of different temperatures (35, 36, 37, 38, 40, and 41 °C) on the RAA-LFD assay was examined. Subsequently, the impact of varying amplification times (5, 10, 15, 18, 20, and 25 min) on the RAA-LFD assay was examined at the optimal primer concentration and reaction temperature. Optimal reaction conditions (temperature/amplification times) were identified via LFD band analysis. DNA extracted from *S.* pullorum an*d S.* typhimurium (10^7^ CFU/mL) served as the target templates throughout the study, with nuclease-free ddH₂O as a negative control.

### 2.6 Specificity of the dual RAA-LFD assay

Specificity assessments for the dual RAA-LFD assay were performed under optimized conditions with genomic DNA templates from three *S.* pullorum isolates, three *S.* typhimurium strains, and five unrelated bacterial species (Table 1). Templates were prepared by boiling extraction, with nuclease-free ddH₂O serving as the negative control.

### 2.7 Detection sensitivities of the dual RAA-LFD assay

To evaluate the detection sensitivities of the dual RAA-LFD assay, serial 10-fold dilutions were prepared for *S.* pullorum BNCC273132 (ranging from 2.37 × 10^7^ CFU/mL to 2.37 × 10^0^ CFU/mL) and *S.* typhimurium ATCC14028 (ranging from 5.91 × 10^7^ CFU/mL to 5.91 × 10^0^ CFU/mL). Using boiling, the genomic DNA templates for the dual RAA-LFD assay were then extracted from these pure bacterial cultures. Additionally, serial 10-fold dilutions of purified whole-genome DNA were prepared: *S.* pullorum DNA (ranging from 4.53 × 10^7^ fg/μL to 4.53 × 10^0^ fg/μL) and *S.* typhimurium DNA (ranging from 5.70 × 10^7^ fg/μL to 5.70 × 10^0^ fg/μL), with nuclease-free ddH₂O as a negative control. Detection sensitivity for the dual RAA-LFD assay was established using pure bacterial cultures and serially diluted genomic DNA.

### 2.8 Simulated sample detection

Firstly, both *S.* pullorum and *S.* typhimurium were cultured in LB medium at 37 °C for 16 h to reach the logarithmic phase. Chicken fecal samples were then collected from the Animal Experiment Station of Shihezi University. All chicken fecal samples were free of *S.* pullorum and *S.* typhimurium by multiplex PCR and the traditional biochemical identification method for *Salmonella* (GB 4789.4–2024) (23). Subsequently, collected samples underwent artificial inoculation with graded doses of *S.* pullorum and *S.* typhimurium, ranging from 10^0^ CFU/mL to 10^7^ CFU/mL. Weigh 100–200 mg of the sample and resuspend it in 400 μL of lysis buffer. Incubate on ice for 10 min, then centrifuge at 10,000 rpm for 1 min. Add 400 μL of squishing buffer and 20 μL of proteinase K (20 mg/mL), followed by incubation at 65 °C for 30 min to facilitate cell lysis and degradation of nucleases. Following whole-genome DNA extraction via the boiling method, the purified DNA was analyzed by RAA-LFD for simultaneous detection of *S.* pullorum and *S.* typhimurium, using nuclease-free ddH₂O as the negative control.

### 2.9 Detection of clinical samples

Thirty-two chicken fecal samples with suspected *Salmonella* infection were collected from Xinjiang to evaluate the clinical applicability. Following the protocols outlined in the Chinese national standard (GB4789.4–2024), biochemical identification and analysis were conducted on isolates of *S.* pullorum and *S.* typhimurium. For molecular analysis, each sample (10–20 g) was aerobically enriched in LB broth (2 mL, 37 °C, 8 h). After the sample was thermally cleaved with proteinase K, Genomic DNA was then extracted via boiling and analyzed by multiplex PCR, RAA, and dual RAA-LFD assays, with nuclease-free ddH₂O as a negative control. The detection accuracy of dual RAA-LFD detection was simultaneously validated against multiplex PCR and biochemical identification. All samples were run in triplicate.

### 2.10 Data analysis

A one-way ANOVA was performed using IBM SPSS Statistics 20 (IBM, Chicago, IL, United States) to assess the statistical significance of the differences between groups (24). The significance threshold was set at a *p* value of less than 0.05 (25). Cohen’s kappa statistic evaluated agreement between biochemical identification, dual RAA-LFD, and multiplex PCR assays. The criteria were as follows: Kappa coefficient ≥0.75 indicates high consistency, 0.4 ≤ K < 0.75 is moderate consistency, and K < 0.4 is considered poor consistency (25).

## 3 Results

The workflow and principle of dual RAA-LFD determination are divided into three key steps: Firstly, DNA from *S*. pullorum and *S.* typhimurium samples is rapidly extracted by boiling water method. Subsequently, double isothermal amplification was carried out using RAA primers and probes to simultaneously generate biotin /FAM and Dig/FAMRA-labeled DNA amplification products. Finally, the amplification product was diluted 10 times and added to the LFD detection system. This system consists of a sample pad, a binding pad (pre-coated with Au labeled anti-digoxin monoclonal antibody), an absorption pad, a liner and a nitrocellulose filter membrane (containing two test lines T1/T2 and one control line). When the labeled DNA products bind to the colloidal gold antibody on the binding pad, they migrate to the detection area with the solution. The biotin-labeled products are captured by the T1 line (anti-biotin antibody), the DIG-labeled products are captured by the T2 line (anti-Dig antibody), and the unbound colloidal gold particles are intercepted by the control line (anti-rabbit antibody). For negative samples, only the control line shows a red band, while for positive samples, both the test line and the control line show color simultaneously. The visual interpretation of dual targets is achieved through color changes.

### 3.1 Optimal primer screening and dual RAA system validation for *Salmonella* typhimurium and *Salmonella* pullorum detection

**Figure 2:**
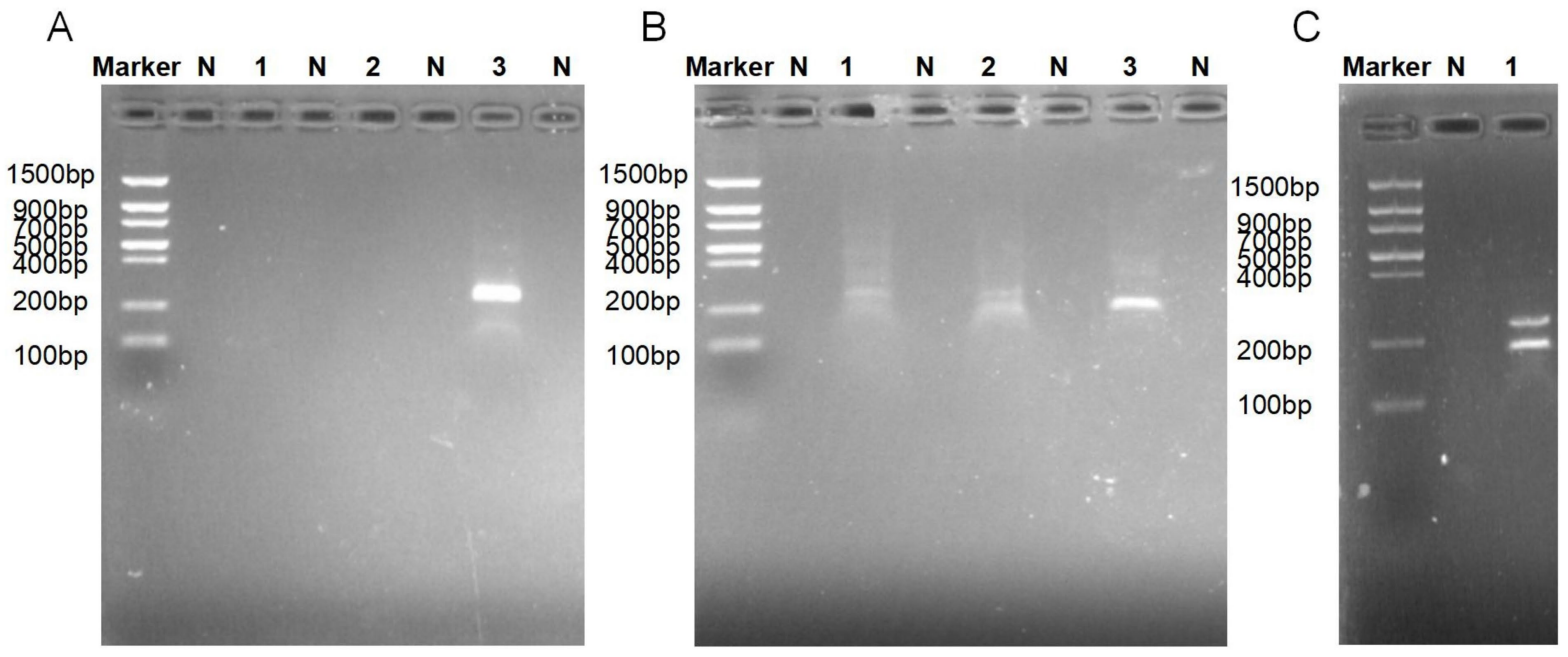
Electrophoretic Validation of Dual RAA Amplification Efficiency. **(A)** Primers screening for *S.* pullorum: Lane1, 2, and 3 were SP-F1/R1, SP-F1/R2 and SP-F1/R3, respectively. **(B)** Primers screening for *S.* typhimurium: Lane 1, 2, and 3 were ST-F1/R1, ST-F2/R2, and ST-F3/R3, respectively. **(C)** RAA amplification: Lane 1 was loaded with a mixture of ST-F3/R3 and SP-F1/R3. SP, *S.* pullorum; ST, *S.* typhimurium; N, negative control, respectively.

Primers in RAA require longer oligonucleotides, typically 30 to 35 bp. Three primer pairs were designed for the *STM4497* gene of *S.* typhimurium and the *ipaj* gene of *S.* pullorum. Among them, maximum amplification efficiency was achieved with ST-F3/R3 and SP-F1/R3 primer sets, as indicated by a single band (Figures 2A,B). RAA amplification yielded target bands of expected sizes, confirming successful establishment of the dual RAA system (Figure 2C). Therefore, these optimal primers were employed to establish the dual-target RAA-LFD assay development.

### 3.2 Optimization of reaction conditions for dual RAA-LFD amplification

**Figure 3:**
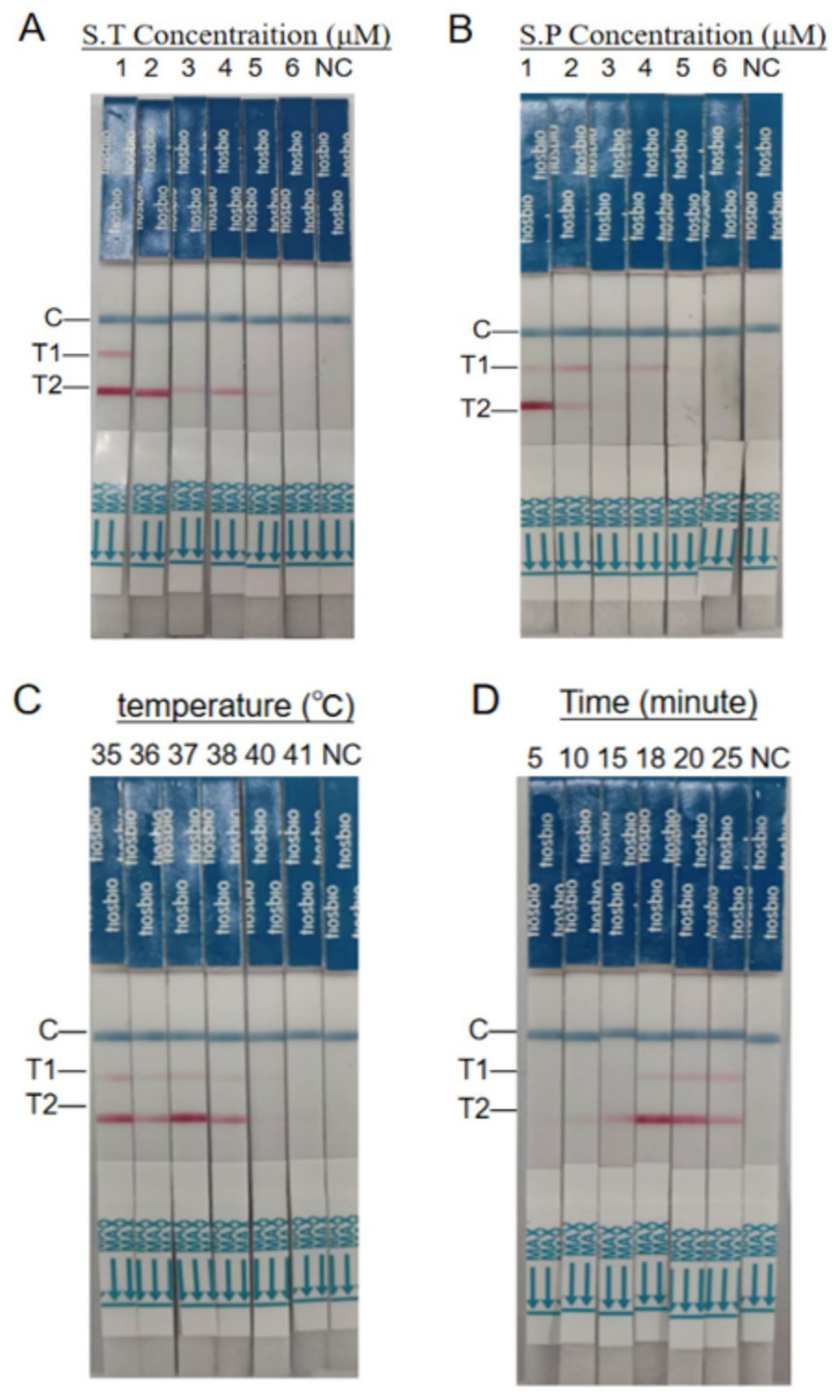
Optimization of dual RAA-LFD reaction conditions. **(A)** Concentration optimization of ST-F3/R3: A primer concentration of 10 μM was used to fix SP-F1/R3, primers ST-F3/R3 with 10 μM, 8 μM, 6 μM, 4 μM, 2 μM, and 1 μM concentration of test strip 1, 2, 3, 4, 5, and 6, respectively. **(B)** Concentration optimization of SP-F1/R3: A primer concentration of 8 μM was used to fix ST-F3/R3, primer SP-F1/R3 with 10 μM, 8 μM, 6 μM, 4 μM, 2 μM and 1 μM concentration of test strip 1, 2, 3, 4, 5 and 6, respectively. **(C)** Optimization of reaction temperatures for ST-F3/R3 and SP-F1/R3. **(D)** Optimization of reaction time for ST-F3/R3 and SP-F1/R3. SP, *S.* pullorum; ST, *S.* typhimurium; T1, *S.* pullorum; T2, *S.* typhimurium; NC, negative control.

Given the potential for cross-interference between primer sets, optimization of both *STM4497* and *ipaj* primer concentrations is necessary in the dual RAA-LFD amplification system. LFD band intensity was visually assessed following 20-min reactions at 38 °C using serially diluted primer concentrations. Non-specific amplification was minimized while amplification efficiency peaked when ST-F3/R3 and SP-F1/R3 concentrations reached 8 μM and 4 μM, respectively (Figures 3A,B). The final RAA reaction component was 50 μL, comprising 25 μL buffer, 0.6 μL each of 10 μM ST/SP-probe, 1.5 μL each of 8 μM ST-F3/R3, and 1.5 μL each of 4 μM SP-F1/R3. 10.8 μL of ddH2O, 2 μL of each *S.* typhimurium and *S.* pullorum DNA template, and 3 μL of an aliquot of Mg(OAc)₂ solution.

Optimal amplification temperatures for RAA-LFD duplex reactions were determined through 20-min metal bath incubations at various temperatures, established 37 °C as the optimal amplification temperature for duplex RAA-LFD reactions (Figure 3C). Optimal reaction time was identified by assessing multiple durations in duplex RAA-LFD amplifications at 37 °C, results demonstrated optimal band intensity and amplification efficiency at 20 min (Figure 3D). Therefore, the optimal RAA-LFD reaction parameters were established as 37 °C for 20 min.

### 3.3 Specificity of the dual RAA-LFD assay

**Figure 4:**
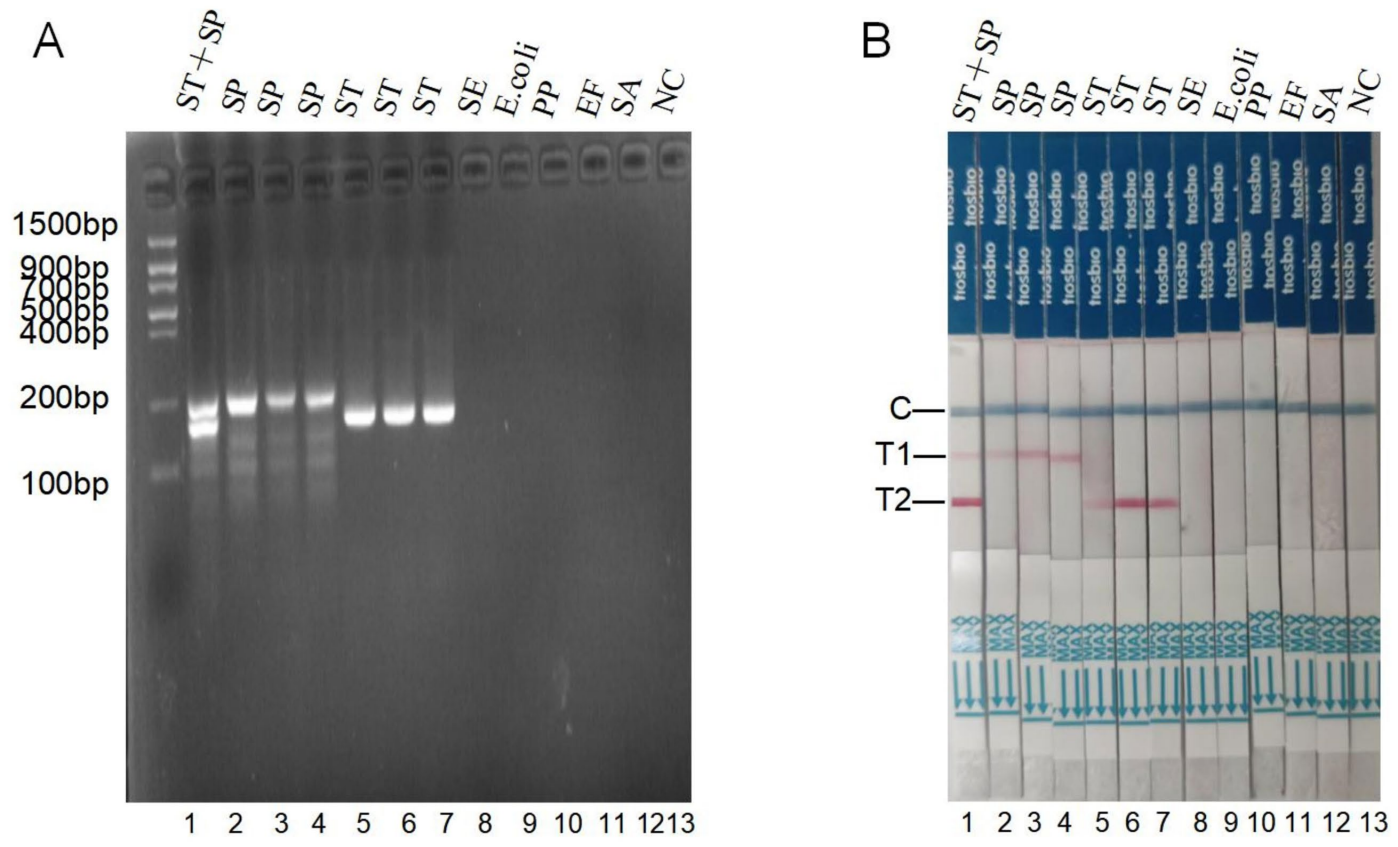
Specificity of the dual RAA assay and dual RAA-LFD assay for *S.* typhimurium and *S.* pullorum in 12 pure bacterial samples. **(A)** Specificity of gel electrophoresis for RAA assay. **(B)** Specificity of dual RAA-LFD assay. ST, *S.* typhimurium; SP, *S.* pullorum; SE, *Salmonella enteritidis*; E.coli, *Escherichia coli*; pp., *Pseudomonas malodulatum*; EF, *Enterococcus fecalis*; SA, *Staphylococcus aureus*; T1, *S.* pullorum; T2, *S.* typhimurium; NC, negative control.

The 12 strains in Table 1 were tested for dual RAA gel electrophoresis display and dual RAA-LFD specificity, respectively, with nuclease-free ddH₂O as a negative control. A clear and unmistakable specific band for *S.* typhimurium and/or *S.* pullorum strains (lanes 1, 2, 3, 4, 5, 6, 7) was a positive result in the RAA assay. In contrast, no specific band for other bacteria (lanes 8, 9, 10, 11, 12) was observed (Figure 4A). The RAA-LFD assay result showed that both double and single positive results for *S.* pullorum and/or *S.* typhimurium showed appropriate test and control lines (strips 1, 2, 3, 4, 5, 6, and 7), while other bacteria tested negative, only control lines appeared in negative controls and with non-target bacteria (Figure 4B). These results indicated that the these two methods have reasonable specificity.

### 3.4 Detection sensitivities of the dual RAA-LFD assay

**Figure 5:**
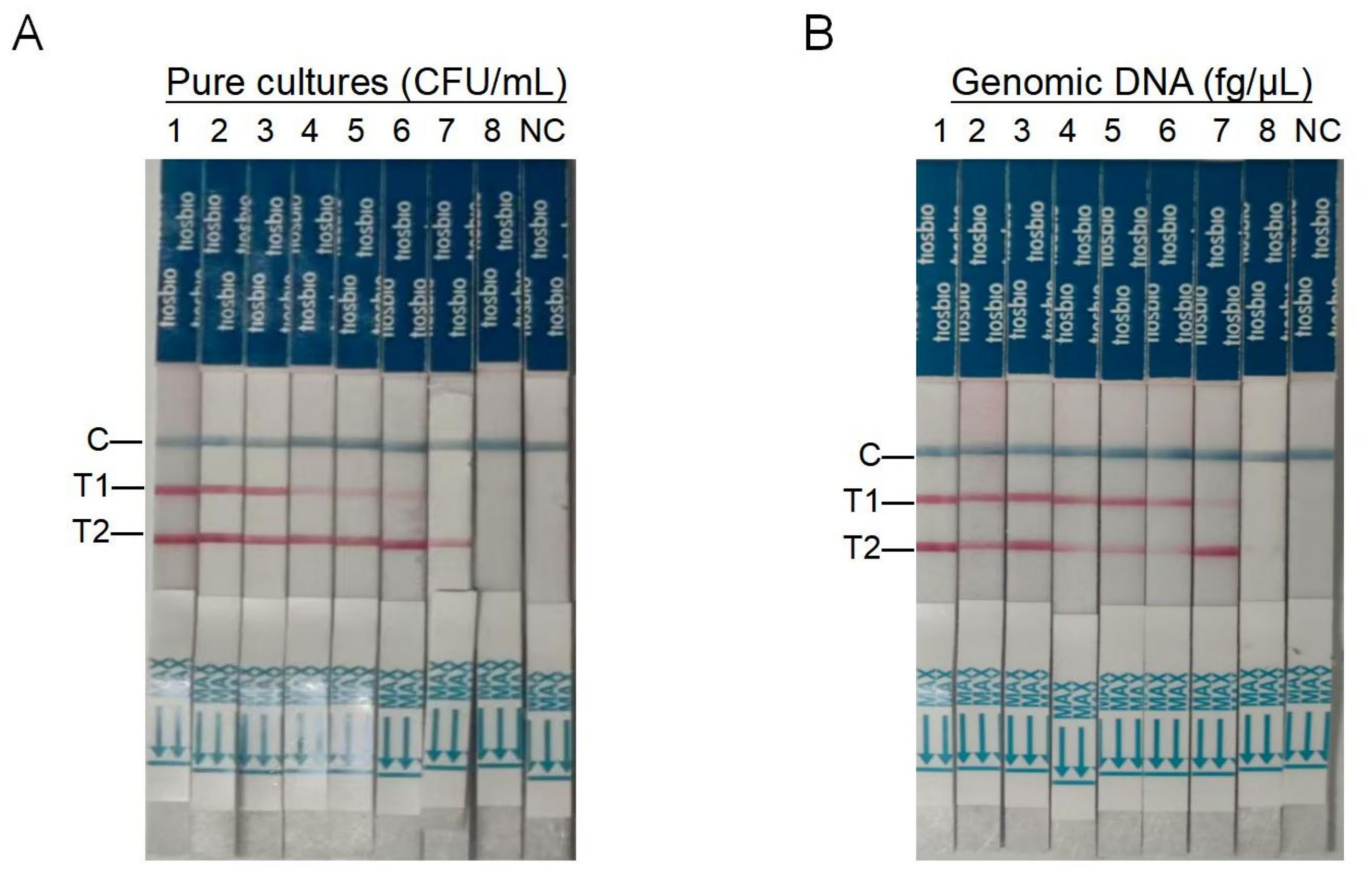
Sensitivity of the dual RAA-LFD for *S.* typhimurium and *S.* pullorum. **(A)** Sensitivity of dual RAA-LFD assay to pure bacterial cultures of *S.* typhimurium and *S. pullorum*; Strips 1 to 8 were *S.* typhimurium-5.91 × 10^7^ CFU/ mL-5.91 × 10^0^ CFU/mL and *S.* pullorum-2.37 × 10^7^ CFU/mL-2.37 × 10^0^ CFU/mL. **(B)** Sensitivity of dual RAA-LFD to whole genome DNA of *S.* typhimurium and *S.* pullorum; The test strips 1–8 were *S.* typhimurium-5.70 × 10^7^ fg/μL to 5.70 × 10^0^ fg/μL and *S.* pullorum-4.53 × 10^7^ fg/μL to 4.53 × 10^0^ fg/μL. T1, *S.* pullorum; T2, *S.* typhimurium; NC, negative control, colony-forming units (CFU).

The dual RAA-LFD detection sensitivities of *S.* pullorum and *S.* typhimurium was evaluated by the pure bacterial concentrations of *S.* typhimurium and *S.* pullorum, which ranged from 5.91 × 10^7^ CFU/mL to 5.91 × 10^0^ CFU/mL and from 2.37 × 10^7^ CFU/mL to 2.37 × 10^0^ CFU/mL, respectively. Visual analysis confirmed that *S.* typhimurium concentrations below 10^1^ CFU/mL failed to produce a detectable T2 test line, while *S.* pullorum required >10^2^ CFU/mL for visible T1 line formation (Figure 5A). Consequently, the duplex RAA-LFD assay demonstrated detection limits of 5.91 × 10^1^ CFU/mL for *S.* typhimurium and 2.37 × 10^2^ CFU/mL for *S.* pullorum under simultaneous detection conditions.

The dual RAA-LFD detection limit of *S.* pullorum and *S.* typhimurium was evaluated by the Genomic DNA concentrations of *S.* typhimurium and *S.* pullorum, which ranged from 5.70 × 10^7^ fg/μL to 5.70 × 10^0^ fg/μL and from 4.53 × 10^7^ fg/μL to 4.53 × 10^0^ fg/μL, respectively. Results demonstrated progressively diminished T-line band intensity on test strips with decreasing genomic DNA concentrations. Crucially, neither T1 nor T2 lines produced detectable bands at 10^0^ fg DNA levels, while distinct test lines appeared at 10^1^ fg DNA concentration. Consequently, detection sensitivities for the dual RAA-LFD assay were established at 5.70 × 10^1^ fg/μL for *S.* typhimurium and 4.53 × 10^1^ fg/μL for *S.* pullorum (Figure 5B).

### 3.5 Application of dual RAA-LFD detection in artificially contaminated samples

**Figure 6:**
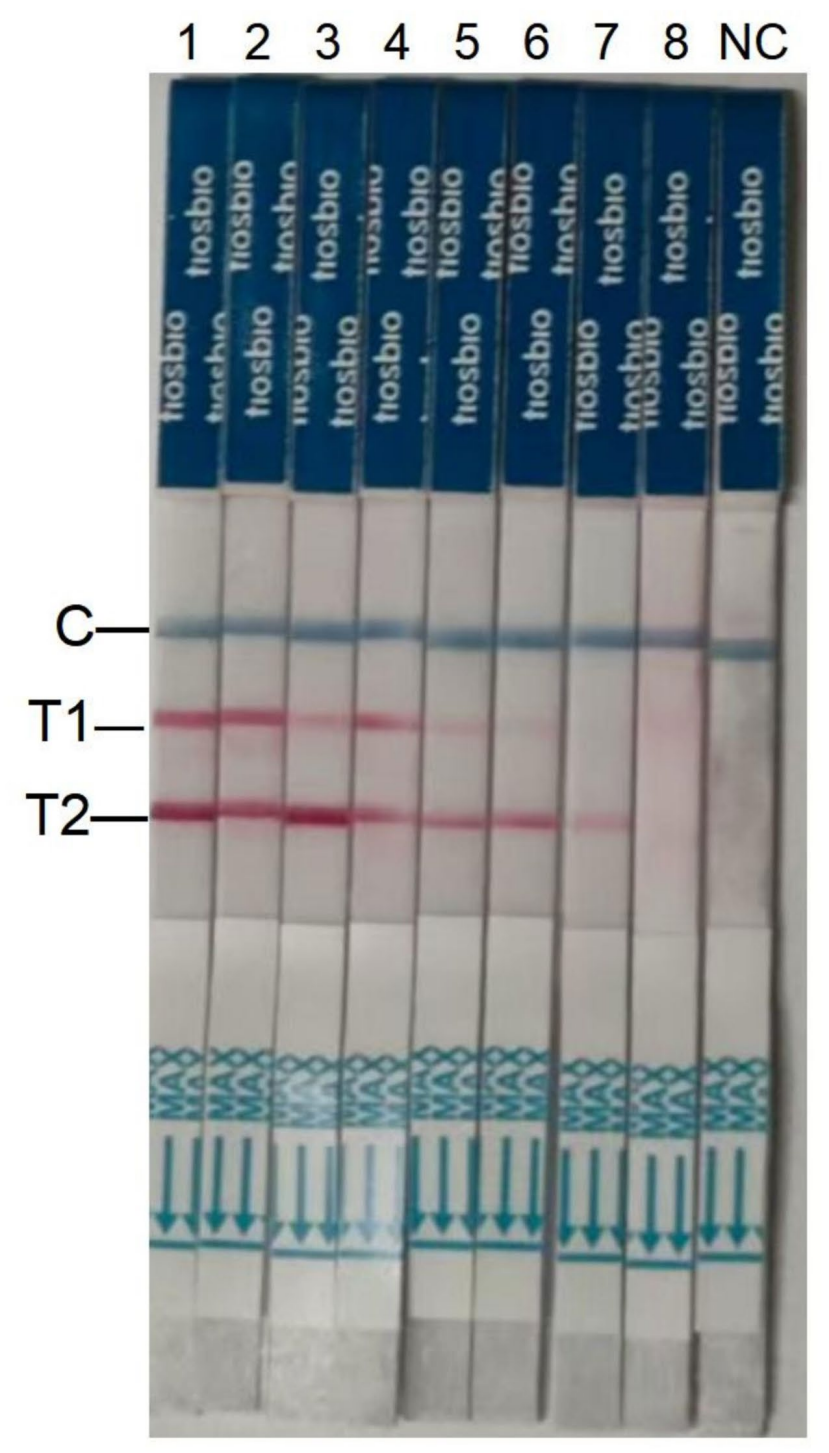
Detection limits of the dual RAA-LFD assay for *S.* typhimurium and *S.* pullorum in pure bacterial samples. For strips 1 to 8, these were *S.* typhimurium-6.26 × 10^7^ CFU/ mL-6.26 × 10^0^ CFU/mL and *S.* pullorum-3.92 × 10^7^ CFU/ mL-3.92 × 10^0^ CFU/mL. T1, *S.* pullorum; T2, *S.* typhimurium; NC, negative control, colony-forming units (CFU).

To evaluate assay applicability, negative fecal samples were spiked with varying concentrations of *S.* typhimurium and *S.* pullorum. Visual analysis revealed undetectable test lines at bacterial concentrations below 10^1^ CFU/mL for *S.* typhimurium and below 10^2^ CFU/mL for *S.* pullorum (Figure 6). Thus, the dual RAA-LFD method demonstrated detection limits of 6.26 × 10^1^ CFU/mL for *S.* typhimurium and 3.92 × 10^2^ CFU/mL for *S.* pullorum in artificially contaminated samples, with values remaining roughly comparable to those in pure cultures.

### 3.6 Clinical sample detection

**Figure 7:**
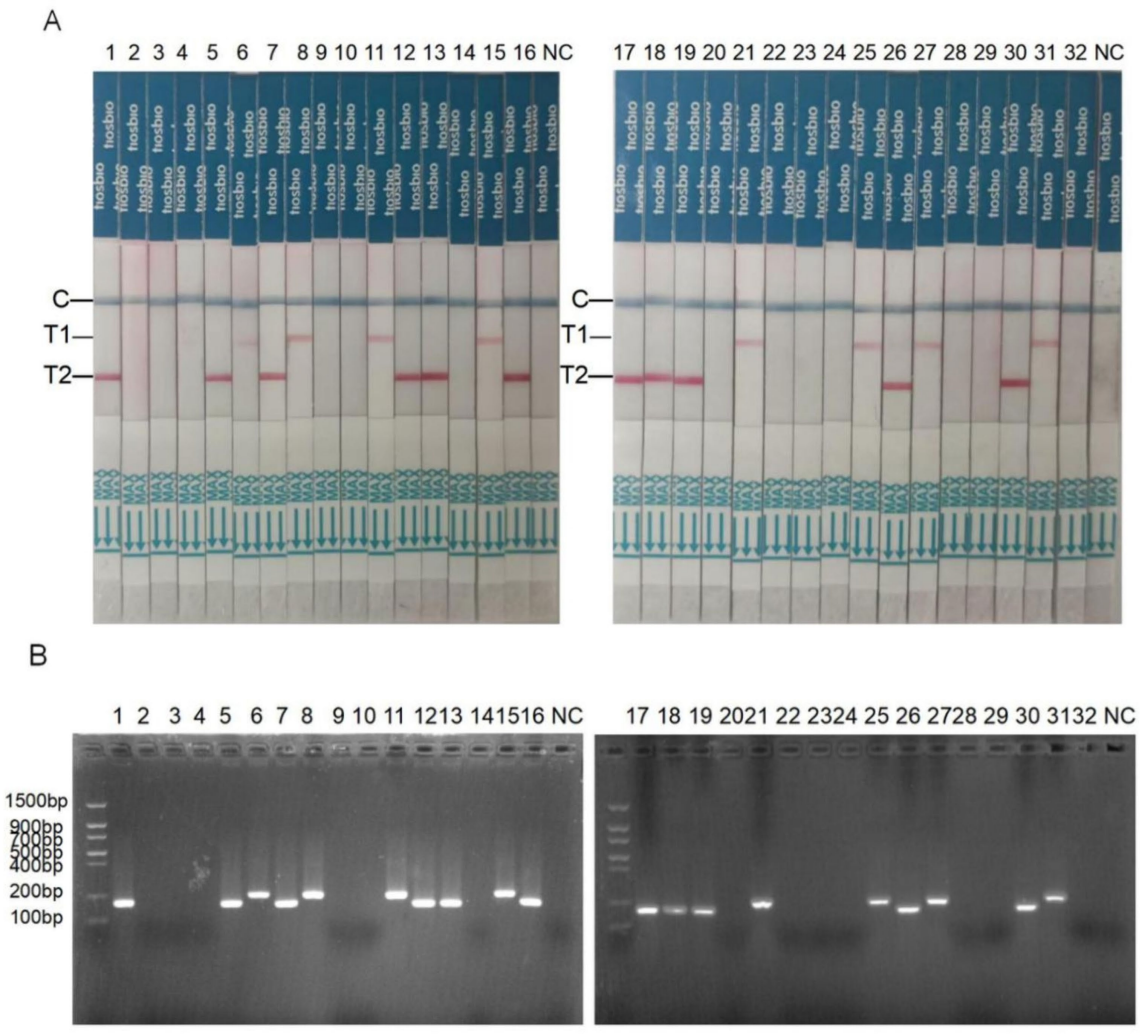
*S.* typhimurium and *S.* pullorum were detected by dual RAA-LFD assay and gel electrophoresis in 32 clinical samples. **(A)** *S.* pullorum and *S.* typhimurium were detected by a dual RAA-LFD assay. **(B)** *S.* pullorum and *S.* typhimurium were determined by gel electrophoresis. T1, *S.* pullorum; T2, *S.* typhimurium; NC, negative control, colony-forming units (CFU).

The dual RAA-LFD and RAA methods detected *S.* typhimurium and *S.* pullorum in 32 clinical chicken fecal samples. The dual RAA-LFD test showed that strips 1, 5, 7, 12, 13, 16, 17, 18, 19, 26, and 30 were positive for *S.* typhimurium, and test strips 6, 8, 11, 15, 21, 25, 27, and 31 were positive for *S.* pullorum. The remaining dipsticks were negative for *S.* typhimurium and *S.* pullorum (Figure 7A). *S.* typhimurium was positive in lanes 1, 5, 7, 12, 13, 16, 17, 18, 19, 26, and 30. Lanes 6, 8, 11, 15, 21, 25, 27, and 31 were *S.* pullorum positive. The remainder were negative for *S.* typhimurium and *S.* pullorum (Figure 7B). This indicated that both dual RAA and dual RAA-LFD test results were identical.

A comparative evaluation of the diagnostic efficacy of the duplex RAA-LFD assay was performed against both biochemical identification (GB 4789.4–2024) and multiplex PCR analysis using fecal specimens. As shown in Table 3, the duplex RAA-LFD assay demonstrated complete concordance with the culture-based method for *S.* typhimurium detection, with both methods identifying all 11 positive samples (Total concordance rate, TCR = 100%; Cohen’s Kappa, K = 1.00). Multiplex PCR exhibited identical performance. For *S.* pullorum detection, the duplex RAA-LFD assay detected 8 positives, whereas both reference methods identified 9 positives, indicating a single false-negative result by the duplex RAA-LFD. Statistical analysis revealed near-perfect agreement between the duplex RAA-LFD and the reference methods for *S.* pullorum (TCR = 96.88%; K = 0.92). All *κ* values were well above the 0.75 threshold, indicating excellent inter-method reliability. The specific results of testing all 32 samples using the three aforementioned methods were presented in Supplementary Table 1.

**Table 3:** Detection of actual samples using dual RAA-LFD assays and multiplex PCR compared to biochemical identification methods.

Strains	*S.* typhimurium		*S.* pullorum	
	Dual RAA-LFD/GB 4789.4–2024	Dual RAA-LFD/ multiplex PCR	Dual RAA-LFD/GB 4789.4–2024	Dual RAA-LFD/ multiplex PCR
Specimens (32)	11/11	11/11	8/9	8/9
TP	11	11	8	8
TN	21	21	23	23
FP	0	0	0	0
FN	0	0	1	1
PPV (%)	100.00	100.00	100.00	100.00
NPV (%)	100.00	100.00	95.83	95.83
Sensitivity (%)	100.00	100.00	88.89	88.89
TCR (%)	100.00	100.00	96.88	96.88
Kappa	1	1	0.92	0.92
P	<0.001	<0.001	<0.001	<0.001

TP, True positive; TN, True negative; FP, False positive; FN, False negative; PPV (Positive predictive value) = TP/(TP + FP) × 100%; NPV (Negative predictive value) = TN/(TN + FN) × 100%; Sensitivity = TP/(TP + FN) × 100%; Specificity = TN/(TN + FP) × 100%; TCR (Total coincidence rate) = (TP + TN)/total sample quantity × 100%; K (Kappa coefficient): K ≥ 0.75 (high consistency); *p* value of less than 0.05 was considered highly significant.

## 4 Discussion

*S.* typhimurium and *S.* pullorum can be transmitted horizontally and vertically and are currently endemic mainly in China and other developing countries where poultry farming is thriving. Its persistent infectious nature makes eradication difficult (26). For such pathogens, efficient detection techniques are the key control means (27). At present, the mainstream detection techniques for pathogens in feces mainly include three categories: Real-time PCR (28), ELISA (29), and electrochemical/optical biosensors (30). The comprehensive application of these detection methods provides important technical support for rapidly screening pathogens. Nevertheless, such techniques prove impractical for large-scale field deployment because they depend on costly instrumentation and labor-intensive protocols (19). With the increase in mixed infection cases, the traditional single-target detection has become insufficient. Therefore, this study developed a dual-target detection method based on RAA-LFD to efficiently identify *S.* typhimurium and *S.* pullorum pathogen DNA in poultry feces in a single reaction system, and its clinical application value was verified.

Isothermal amplification technology achieves nucleic acid amplification under constant temperature conditions, thereby overcoming the dependence of traditional PCR on temperature cycling (31). This feature simplifies testing equipment requirements, especially for point-of-care testing (POCT) and low-resource areas (32). Current mainstream isothermal amplification methods include LAMP, RPA, and RAA (33, 34). Among them, recombinase polymerase amplification (RAA) technology has made a significant breakthrough in recent years. Through optimized primer design and application of novel enzyme components, RAA has shown outstanding detection specificity and sensitivity (35), often combined with lateral flow chromatography or fluorescence detection to achieve rapid visual analysis (36, 37). A dual RAA response can simultaneously detect multiple targets (38), offering critical utility for differential diagnosis when symptoms converge or coinfections occur, thereby informing novel diagnostic pathways for complex microbial presentations.

The core primer design of the *S.* typhimurium and *S.* pullorum dual recombinase-mediated isothermal amplification (RAA) assay system constructed in this study is based on two particular molecular targets: the *STM4497* gene as a specific marker of *S.* typhimurium (39), and the *ipaJ* gene as a molecular diagnostic target of *S.* pullorum (40, 41). We optimized the key parameters of the RAA reaction. Temperature gradient experiments showed that 37 °C was the optimal reaction temperature, which effectively inhibited nonspecific amplification while maintaining the activity of RAA. The 20-min reaction duration not only ensured amplification efficiency but also ensured timeliness. The optimized primer concentrations: At 8 μM ST-F3/R3 and 4 μM SP-F1/R3 concentrations, an optimum balance between sensitivity and specificity was observed. The experimental results showed that the optimized system could efficiently detect two types of *Salmonella* simultaneously, and the amplification efficiency reached its maximum, providing reliable technical support for rapid on-site diagnosis.

This study’s dual RAA-LFD detection method was compared with Multiplex PCR, TaqMan multiplex Real-Time PCR, LAMP, CRISPR/Cas-based biosensors, digital PCR, and Colorimetric assay, as shown in Table 4, the duplex RAA-LFD detection method established in this study demonstrates higher specificity and sensitivity compared to other detection methods, with additional advantages of shorter reaction time and visual detection capability. Critically, this method addresses the high false-positive rate of traditional LAMP by integrating FAM/TAMRA-labeled probes with LFD test strips. Consequently, the entire “sample-to-result” process requires only test strips and a water bath, while maintaining detection sensitivities of 6.26 × 10^1^ CFU/mL (*S.* typhimurium) and 3.92 × 10^2^ CFU/mL (*S.* pullorum) in simulated fecal samples-making it suitable for grassroots field detection. Economically, the duplex RAA-LFD reduces reagent consumption by 50% versus single-assay systems, with enzyme costs 50% lower than RPA.

**Table 4:** Comparison between this study and previous *S.* pullorum and *S.* typhimurium assays.

Sample type	Sample preparation time (min)	Methods	Limit of detection	Specificity	Sensitivity	Time of detection (min)	Visual detection	Ref.
Dead chicken embryo	90–120	Multiplex PCR	10^2^ CFU/mL	100%	100%	150	NO	(23)
liver samples	60–90	TaqMan Multiplex Real-Time PCR	10^1^ CFU/mL	100%	100%	100	NO	(43)
poultry carcasses	60	LAMP	10^1^ CFU/mL	100%	—	60	YES	(44)
meat	80	Digital PCR	90 CFU/reaction	—	94.5%	120	NO	(45)
pure culture	15–20	CRISPR/Cas-based biosensors	7.9 × 10^1^ CFU/reaction	—	—	120	NO	(42)
meat	60–80	photonic PCR-LFIS	10^2^ CFU/mL	—	84%	80	YES	(46)
water	35	Colorimetric biosensors	7 CFU/mL	—	—	40	YES	(47)
feces	35	Dual RAA-LFD(ST)	6.26 × 10^1^ CFU/mL	100%	100%	20	YES	This study
feces	35	Dual RAA-LFD(SP)	3.92 × 10^2^ CFU/mL	100%	88.89%	20	YES	This study

ST, *S*. typhimurium; SP, *S*. pullorum; CFU, colony-forming unit.

The current methods also have some drawbacks. We detected one false negative case of *S.* pullorum in 32 clinical samples. The large amount of microorganisms, undigested food residues, and metabolic products contained in feces may interfere with the efficiency of nucleic acid extraction, leading to false-negative results. Meanwhile, this may be because the ipaj gene is not present in all *S.* pullorum. Among the 650 *S.* pullorum strains isolated in China from 1962 to 2016, a total of 644 plasmid pSPI12 strains carrying the ipaJ gene were identified, and six false-negative *S.* pullorum disease strains were detected (40). False-positive results may also be due to the inactivation of the buffer, repeated freezing and thawing, or improper operation, leading to a decrease in enzyme activity and thereby reducing amplification efficiency. However, prolonged exposure to air may impair the stability of the double RAA-LFD strip, thereby increasing the risk of false-positive results (42). Thus, results should be interpreted within 30 min of testing. At the same time, indoor ventilation should be maintained to reduce the contamination of nucleic acids. Moreover, the DNA of non-target microorganisms may compete for primers or enzyme resources, resulting in a decrease in amplification efficiency (48). Therefore, sample processing before product testing is of great significance. It is very important to improve the efficiency of sample processing. When the number of samples is large, the nucleic acid extraction process for these samples is very time-consuming. In addition, although nucleic acid test strips have the advantages of being fast and convenient, their qualitative detection characteristics limit their application in scenarios that require quantitative analysis (18). Rigorous evaluation of the dual RAA-LFD assay through comparative analysis of artificially contaminated and clinical samples against multiplex PCR and biochemical identification. The platform consistently demonstrated high sensitivity and specificity, supporting its utility as a rapid and efficient pathogen detection system.

## 5 Conclusion

This study successfully developed and validated a novel RAA-LFD assay for the rapid, specific, and sensitive point-of-care detection of *S.* pullorum and *S.* typhimurium. Targeting the *ipaj* (*S.* pullorum) and *STM4497* (*S.* typhimurium), the assay operates at a constant 37 °C and delivers visual results within 20 min. Collectively, these findings, in this study, confirm the precision, high sensitivity, and robustness of the dual RAA-LFD assay. By eliminating the need for sophisticated instrumentation and complex thermal cycling, this technology provides a powerful, user-friendly point-of-need screening tool. Its speed, simplicity, and visual readout make it ideally suited for grassroots-level field deployment, offering significant potential for early detection and containment of avian salmonellosis outbreaks and enhancing food safety surveillance throughout the poultry industry.

## Data Availability

The original contributions presented in the study are included in the article/Supplementary material, further inquiries can be directed to the corresponding authors.
