# Supplementary material for: Development of a dual-target RAA-LFD assay for point-of-care and visual detection of Salmonella pullorum and Salmonella typhimurium in fecal samples
Source: Front Vet Sci. 2025 Nov 3;12:1684537. doi: 10.3389/fvets.2025.1684537 (PMC12621324; doi:10.3389/fvets.2025.1684537)
Supplement: Supplementary file 1 [file Table_1.docx]

Supplementary Material

**Supplementary Table 1. Clinical sample testing by three methods**

| Strains/Specimens (32) | *S.* typhimurium | | | *S.* pullorum | | |
| --- | --- | --- | --- | --- | --- | --- |
|  | Dual RAA-LFD | Multiplex PCR | Biochemical identification | Dual RAA-LFD | Multiplex PCR | Biochemical identification |
| 1 | + | + | + | — | — | — |
| 2 | — | — | — | — | — | — |
| 3 | — | — | — | — | — | — |
| 4 | — | — | — | — | — | — |
| 5 | + | + | + | — | — | — |
| 6 | — | — | — | + | + | + |
| 7 | + | + | + | — | — | — |
| 8 | — | — | — | + | + | + |
| 9 | — | — | — | — | — | — |
| 10 | — | — | — | — | — | — |
| 11 | — | — | — | + | + | + |
| 12 | + | + | + | — | — | — |
| 13 | + | + | + | — | — | — |
| 14 | — | — | — | — | — | — |
| 15 | — | — | — | + | + | + |
| 16 | + | + | + | — | — | — |
| 17 | + | + | + | — | — | — |
| 18 | + | + | + | — | — | — |
| 19 | + | + | + | — | — | — |
| 20 | — | — | — | — | + | + |
| 21 | — | — | — | + | + | + |
| 22 | — | — | — | — | — | — |
| 23 | — | — | — | — | — | — |
| 24 | — | — | — | — | — | — |
| 25 | — | — | — | + | + | + |
| 26 | + | + | + | — | — | — |
| 27 | — | — | — | + | + | + |
| 28 | — | — | — | — | — | — |
| 29 | — | — | — | — | — | — |
| 30 | + | + | + | — | — | — |
| 31 | — | — | — | + | + | + |
| 32 | — | — | — | — | — | — |
